# Strangulation de la verge par un anneau chez l’enfant

**DOI:** 10.11604/pamj.2016.25.173.11112

**Published:** 2016-11-17

**Authors:** Abdoulaye Diallo Harouna, El Madi Aziz

**Affiliations:** 1 Service de Chirurgie Pédiatrique Viscérale et Urologique CHU Hassan II, Fès, Maroc

**Keywords:** Strangulation, verge, anneau, enfant, Strangulation, penis, ring, child

## Image en médecine

Enfant de 8 ans sans antécédent pathologique particulier, qui a été amené aux urgences pédiatriques par ses parents pour un fait inédit, une strangulation de la verge remontant 5 jours auparavant. Le patient avait curieusement introduit sa verge dans un anneau. L'enfant avait caché les faits par peur et pudeur avec l'espoir de pouvoir l'extraire seul. Devant l'installation progressive d'une douleur et une tuméfaction du gland avec l'impossibilité de pouvoir retirer l'anneau, le patient était contraint d'avouer à ses parents qui étaient embarrassés et furieux. A l'admission, il était conscient, sans trouble psychique ni de comportement. L'examen retrouvait un important œdème de la verge en aval d'un anneau fermé très constrictif avec une bonne viabilité du gland (A). Le geste a consisté à une ablation de l'anneau par cisaillement à l'aide d'une pince coupante (B), après une protection préalable de la verge. Après une semaine on note régression complète de l'œdème sans signes urinaires (C).

**Figure 1:**
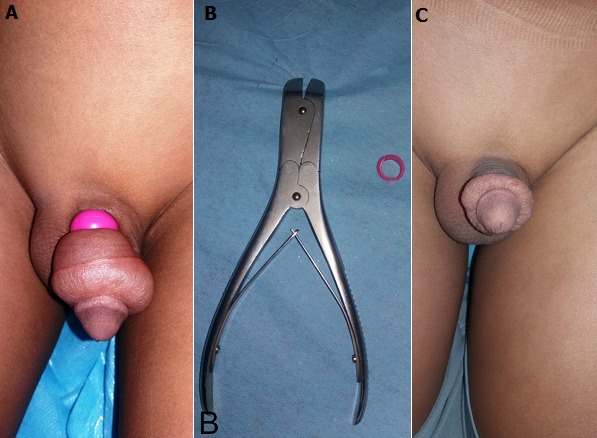
A) strangulation de la verge par un anneau fermé très constrictif; B) pence coupante (ou coupe broche); C) régression de l’œdème, sans signes urinaires

